# Process Optimization on Trepanning Drilling in Titanium Alloy Using a Picosecond Laser via an Orthogonal Experiment

**DOI:** 10.3390/mi16080846

**Published:** 2025-07-24

**Authors:** Liang Wang, Yefei Rong, Long Xu, Changjian Wu, Kaibo Xia

**Affiliations:** 1 Faculty of Mechanical and Materials Engineering, Huaiyin Institute of Technology, Huai’an 223003, China; wangliang@hyit.edu.cn (L.W.); 19816090517@163.com (L.X.); jianjian56791116@163.com (C.W.); 2 School of Mechanical Engineering, Jiangsu University, Zhenjiang 212013, China

**Keywords:** picosecond laser, titanium alloy, laser drilling, orthogonal experimental design, process optimization

## Abstract

To optimize the laser drilling process and reduce the processing time, this study investigates picosecond laser trepan drilling on the titanium alloy TC4, analyzing the effects of laser parameters on micro-hole diameter, taper, and roundness. Four independent variables were selected: laser power, defocusing distance, scanning speed, and the number of scans. An L_25_ (5^6^) orthogonal array was employed for experimental design. The mean response and range analyses evaluated parameter impacts on micro-hole quality, revealing the influence mechanisms of these variables at different levels. The results indicate the following: (1) the scanning speed and laser power significantly affect entrance and exit micro-hole diameters; (2) the defocusing distance substantially influences micro-hole taper; (3) the laser power most critically impacts inlet roundness; (4) the defocusing distance, scanning speed, and laser power directly correlate with outlet roundness; (5) the number of scans exhibits weaker relationships with inlet/outlet diameters, taper, and roundness. A comprehensive balance method applied to orthogonal test results for process optimization yielded the following optimal parameters: 90% laser power (30 W total), −0.2 mm defocus, a 27 mm/s scanning speed, and 15 scans.

## 1. Introduction

**Figure 1:**
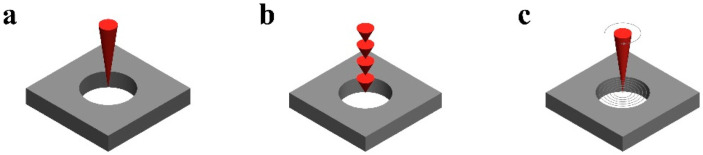
Schematic diagram of laser drilling hole: (**a**) single-pulse drilling; (**b**) multi-pulse drilling; (**c**) trepan drilling.

Titanium alloys are composed primarily of titanium with alloying elements such as aluminum, vanadium, and molybdenum. These properties enable widespread applications in the aerospace, military, chemical, and biomedical sectors [1]. In the aerospace industry, for example, the performance and reliability of gas turbine engines heavily depend on the quality of micro-scale cooling holes drilled into turbine components to prevent overheating [2]. In the biomedical sector, the long-term success of surgical implants is often improved by creating micro-porous structures on their surfaces, which facilitate bone ingrowth and enhance biocompatibility [3]. Unfortunately, machining these micro-features in TC4 is notoriously challenging. Its high hardness and low thermal conductivity mean that traditional mechanical drilling is often inadequate, leading to issues like rapid tool wear, poor surface finish, and thermal damage, failing to meet the required precision level [4,5,6]. As an advanced material processing method, laser drilling reduces tool wear and achieves higher processing precision than traditional mechanical drilling methods [7]. Based on processing requirements and material characteristics, laser drilling methods are classified into three categories, as shown in Figure 1: single-pulse drilling, multi-pulse drilling, and trepanning drilling [8,9,10,11]. Single-pulse drilling employs a single prolonged laser exposure to create holes. Multi-pulse drilling utilizes sequential laser pulses to induce thermal shock, enabling rapid hole formation. Trepanning drilling involves multiple laser scans along predefined paths, with each pass incrementally deepening the hole to achieve target dimensions. By optimizing scan trajectories and repetition counts, this method ensures precise control over hole geometry and enhanced processing accuracy [12].

Laser drilling is a prevalent technique used for fabricating high-quality micro-holes in TC4 titanium alloy. To this end, researchers have explored various methods for enhancing machining outcomes. For instance, S. Amiri et al. investigated the influence of concurrently applied ultrasonic vibrations and magnetic fields on the quality of TC4 [13]. K. A. I. F. B. Ku et al. examined the effect of laser looping strategies, achieving improved surface finish and reduced roughness [14]. M. Saravanan et al. employed an orthogonal experimental design to optimize laser power, frequency, and scanning speed, thereby enhancing the roundness of micro-holes [15]. Similarly, P. Deepu et al. utilized a femtosecond laser to drill TC4, focusing on the combined effects of process parameters and scanning strategies for micro-hole characteristics [16]. VK Bupesh Raja et al. integrated an Artificial Neural Network (ANN) with an orthogonal experimental method to determine the optimal parameters for hole roundness in laser-drilled TC4 [17]. Consequently, while substantial research exists on the laser drilling of titanium alloys, the optimization of picosecond laser drilling processes for TC4 and the enhancement of micro-hole quality warrant further investigation. Specifically, there is a scarcity of studies focusing on the synergistic effects of multiple laser parameter combinations on the overall quality characteristics of micro-holes.

This study uses an orthogonal experimental design for aTC4 titanium alloy to conduct picosecond laser trepanning drilling experiments. Laser parameters—including laser power, defocusing distance, the number of scans, and scanning speed—are systematically varied. Range analysis quantify the effects of these parameters on micro-hole quality metrics: entrance/exit diameters, entrance/exit roundness, and taper. The optimal process parameter set is subsequently determined. This study aims to provide a robust and optimized process window based on a reliable orthogonal experimental design, which can serve as a valuable and practical guide for industrial applications.

## 2. Experimentation Details

### 2.1. Materials and Equipment

**Figure 2:**
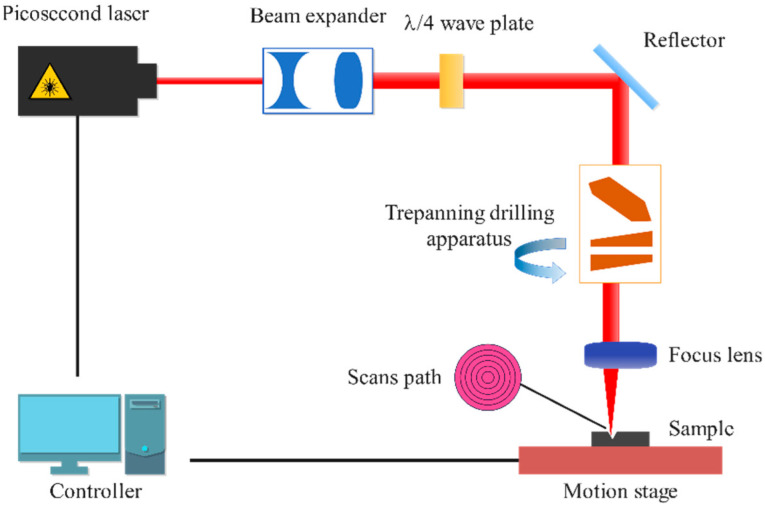
Laser drilling device.

The experiments employed a high-precision picosecond laser processing system with three core subsystems: a laser source, beam delivery optics, and a precision positioning stage, as shown in Figure 2. An OLYMPUS DXS100 3D laser scanning microscope was utilized for micro-hole morphology characterization.

The experimental material was a titanium alloy TC4 disc (30 mm diameter × 0.5 mm thickness), whose chemical composition is detailed in Table 1.

### 2.2. Experimentation Program

This study employed a robust orthogonal design methodology to investigate and optimize the process parameters for micro-hole drilling systematically. The selection of parameter ranges was based on preliminary experiments that identified a promising processing window for four key variables: laser power (A), defocusing distance (B), scanning speed (C), and the number of scans (D), as ranges outside this window were found to yield undesirable outcomes such as incomplete penetration or excessive thermal damage. The experimental plan was structured using an L_25_ (5^4^) orthogonal array, assigning five discrete levels to each factor, as detailed in Table 2. Critical system parameters were held constant throughout all trials to ensure scientific rigor and reproducibility: the pulse repetition rate was fixed at 1000 Hz (1 kHz) and the focused beam diameter was fixed at 16 µm. Consequently, the laser power, which was varied from 75% to 95% of a 30 W maximum, corresponded to a single-pulse energy range of 22.5 mJ to 28.5 mJ. This overall approach enabled an efficient investigation with minimal trials while ensuring the statistical adequacy of the collected data [18,19].

**Figure 3:**
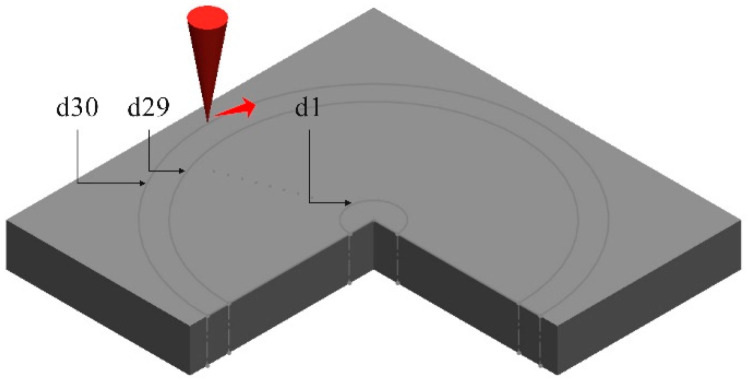
Scanning the path diagram. The red arrow indicates the scanning direction.

Figure 3 illustrates the concentric-circle scanning path strategy, progressing inward to outward. The innermost circle diameter was d1 = 0.010 mm, with successive circles increasing by 0.010 mm increments, culminating in the outermost circles, i.e., d29 = 0.290 mm and d30 = 0.300 mm.

### 2.3. Quality Assessment Methods

**Figure 4:**
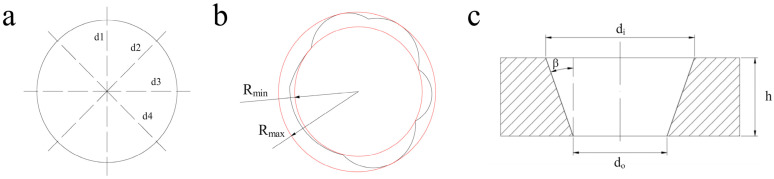
Micro-hole feature characterization: (**a**) hole diameter; (**b**) hole roundness; (**c**) through-hole taper.

To minimize measurement uncertainty, each micro-hole was measured at multiple angular positions, as shown in Figure 4a, with adjacent measurement axes separated by 45°. The final diameter was calculated as the arithmetic mean of four independent measurements:

(1) $$d=(d_{1}+d_{2}+d_{3}+d_{4})/4$$

Figure 4b illustrates the roundness measurement methodology for micro-holes. The black contour represents the measured hole profile, while the red circumscribed and inscribed circles denote the maximum (*R*ₘₐₓ) and minimum (*R*ₘᵢₙ) radii positions, respectively. The roundness value (Δ*R*) is defined as

(2) $$\Delta R=R_{\min}-R_{\max}$$

where a smaller ΔR indicates higher geometric regularity.

The micro-hole taper (*β*) was calculated by modeling the through-hole as a truncated cone, as shown in Figure 4c, where *d*_i_ is the entrance hole diameter, *d*_o_ is the exit hole diameter, and *h* is the hole depth. The calculation formula was

(3) $$\beta =\arctan(\frac{d_{i}-d_{o}}{2h})$$

### 2.4. Process Optimization Methods

#### 2.4.1. Range Analysis

The range analysis method was employed to interpret the orthogonal experiment results. This process involves calculating the mean response value (*k*) for each factor at each of its levels. Subsequently, the range of effect (*R*) for each factor was determined by the difference between its maximum and minimum mean response values, as expressed in Equation (4):

(4) R=max{k1,k2,k3,k4,k5}−min{k1,k2,k3,k4,k5}

The magnitude of the range (R) was used to rank the factors according to their degree of influence on the target metric. A larger R value signified that the corresponding factor had a greater impact, whereas a smaller R value indicated a lesser effect.

#### 2.4.2. Comprehensive Balancing Method

For the final analysis of the experimental results, a comprehensive balancing method was adopted. The core principle of this approach is first to analyze each performance metric individually to determine the rank of influencing factors and the optimal parameter combination for that specific metric. Subsequently, these individual findings are holistically integrated through a systematic process to establish a final, optimized parameter configuration. The particular decision-making rules are as follows:

(1) If a factor’s rank of influence varies across different metrics, priority is given to the factor level that is optimal for the metric on which it has the most significant impact;
(2) If a factor exhibits a low overall influence across all metrics, its optimal level is selected based on a “majority rule” principle, choosing the level that appears most frequently as the optimum in the individual analyses;
(3) When the differences in influence among various factors are indistinct, certain less impactful factors may be excluded to avoid redundancy and resource waste, thereby defining a more rational and efficient parameter set;
(4) If a factor has a pronounced effect on a particularly critical performance metric, its optimal level should be determined primarily to satisfy the requirements of that key metric.

## 3. Results

### 3.1. Hole Diameter Analysis

Table 3 lists the average values and range (R) changes in the entrance diameter under five levels of four process parameters: laser power, defocusing distance, scanning speed, and the number of scans. Table 4 shows the changes in the exit diameter.

**Figure 5:**
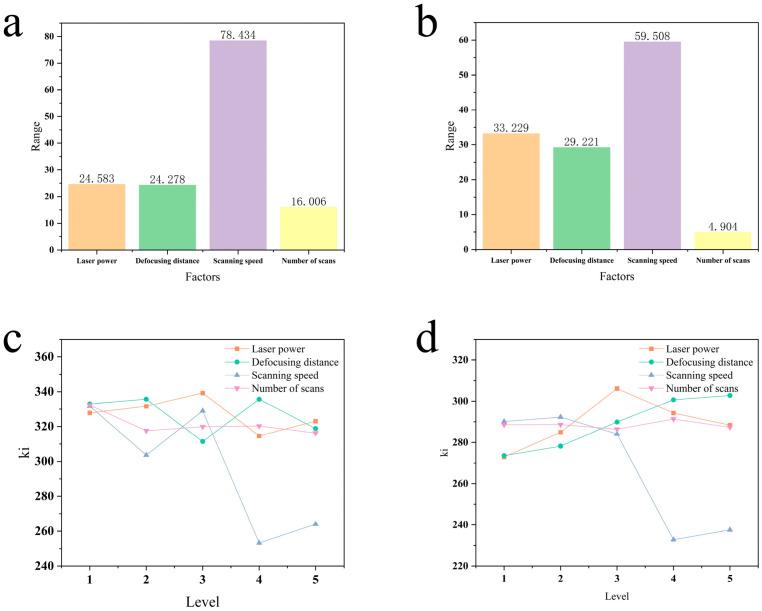
Hole diameter analysis: (**a**) range of entrance diameter; (**b**) range of exit diameter; (**c**) effect of different levels on entrance diameter; (**d**) effect of different levels on exit diameter.

To more intuitively present the strength of the influence of various factors on the aperture size, this paper plots the corresponding entrance diameter and extreme difference average response analysis diagram, as shown in Figure 5a. The order of strength of the correlation between the four factors and the entrance aperture size is C (scanning speed) > A (laser power) > B (defocusing distance) > D (number of scans).

Figure 5c illustrates the trends in the entrance diameter as a function of varying factor levels. The results indicate that the number of scans has a minimal effect on the entrance diameter, a finding that corroborates the conclusions from the range analysis. In contrast, scanning speed exerts a more pronounced influence. As the scanning speed increases, the entrance diameter exhibits a distinct decreasing trend. This is primarily because the scanning speed directly governs the linear energy density—the cumulative laser energy delivered per unit length. A lower scanning speed leads to a sharp increase in accumulated energy, causing more extensive material ablation and, thus, a larger entrance diameter. Conversely, increasing the scanning speed substantially reduces the linear energy density, resulting in a more localized ablation zone, a smaller heat-affected zone, and, consequently, a smaller diameter. The effects of laser power and defocusing distance are comparable. Both factors indirectly influence the hole formation mechanism by modulating the thermal input and the spatial distribution of laser energy. Operating within an appropriate range for these parameters helps to suppress the formation of huge diameters, thereby enhancing machining precision. Therefore, considering the entrance diameter alone, the optimal parameter combination is identified as A_4_B_3_C_4_D_5_.

Similarly, an average response analysis was conducted for the export diameter, and the results are presented in Figure 5b, which compares the effects of the four factors to evaluate their relative influence. The strength of the correlation between each factor and the export diameter is ranked as follows: C (scanning speed) > A (laser power) > B (defocusing distance) > D (number of scans).

Figure 5d shows the trends in the exit diameter as a function of varying factor levels. As shown, scanning speed exerts the most significant control over the exit diameter, which exhibits a generally decreasing trend. This is because a lower scanning speed allows more laser pulses to interact with the exit region; the accumulated energy is sufficient to ablate the material, forming a larger exit region effectively. Conversely, an excessively high scanning speed results in insufficient energy delivery, as the laser beam’s energy is substantially attenuated before it fully penetrates the material, thus hindering effective ablation at the exit. The influence of laser power is non-linear: the diameter increases markedly as the power is increased from 75% to 85% but then decreases slightly with further power increases. This is primarily attributed to the plasma shielding effect. In this well-documented phenomenon, a dense plasma plume forms and absorbs or scatters the energy of subsequent laser pulses, preventing it from reaching the workpiece effectively [20]. The trend for the defocusing distance indicates that positioning the focal point beneath the material surface facilitates the convergence of energy at the exit plane, thereby producing a larger exit. Finally, the number of scans has a minimal impact. This is because once the channel is fully penetrated, the exit morphology is primarily established. The energy from subsequent scans suffers from significant attenuation as it travels through the deep hole and is further impeded by brutal debris expulsion, making it unable to widen the exit diameter significantly. From the perspective of the exit diameter, a comprehensive analysis of all factors leads to the optimal combination of A_1_B_1_C_4_D_3_.

### 3.2. Hole Taper Analysis

Table 5 shows the average response and range analysis table for the experimental indicator taper, listing the average values and range (*R*) changes in the taper at five levels of four process parameters.

**Figure 6:**
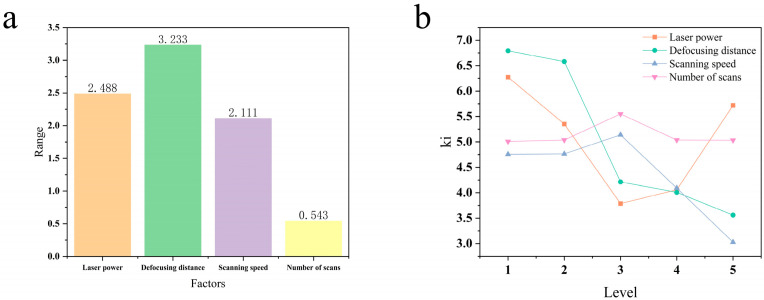
Hole taper analysis: (**a**) range of taper; (**b**) effect of different levels on taper.

To more directly illustrate the influence of the four factors on hole taper, this study presents a bar chart of the extreme taper values under each factor (see Figure 6a). The defocusing distance exhibits the most significant effect, with a range of approximately 3.233°, followed by laser power (2.488°) and scanning speed (2.111°); scanning frequency has the smallest range at about 0.543°. These results indicate that the defocusing distance dominates taper control, whereas the number of scans has a comparatively minor impact. The correlation strength ranking between each factor and entrance taper is B (defocusing distance) > A (laser power) > C (scanning speed) > D (number of scans).

Figure 6b illustrates the trends in hole taper as a function of varying factor levels. As shown in the figure, the defocusing distance exerts the most pronounced influence. As it increases from −0.2 mm to +0.2 mm, the taper exhibits a steady downward trend, with the minimum value observed at +0.2 mm; this suggests that a suitable positive defocus optimizes the energy delivery towards the hole’s tip, potentially by compensating for in-hole beam divergence and leveraging multiple reflections, which can lead to an elevated absorbed fluence at the bottom of the hole [21]. Insufficient power leads to poor penetration, creating a large entrance-to-exit diameter ratio and, thus, a high taper. Conversely, excessive power generates a dense plasma plume within the hole that shields and absorbs subsequent laser energy, concentrating energy deposition in the upper section and again increasing the taper. Therefore, the minimum taper is achieved only at a moderate power level that balances effective machining with minimal plasma shielding. In contrast, the influence of scanning speed is relatively minor in the 18–24 mm/s range, showing a gentle variation, followed by a decreasing trend between 24 mm/s and 30 mm/s. The number of scans has a negligible effect on taper throughout the entire experimental range of 15 to 55 passes, with minimal fluctuation. From the perspective of taper, a comprehensive analysis of all factors identifies the optimal combination as A_3_B_5_C_5_D_1_.

### 3.3. Hole Roundness Analysis

Table 6 and Table 7 show the average values and ranges (*Rs*) of inlet roundness and outlet roundness under five levels of four factors.

**Figure 7:**
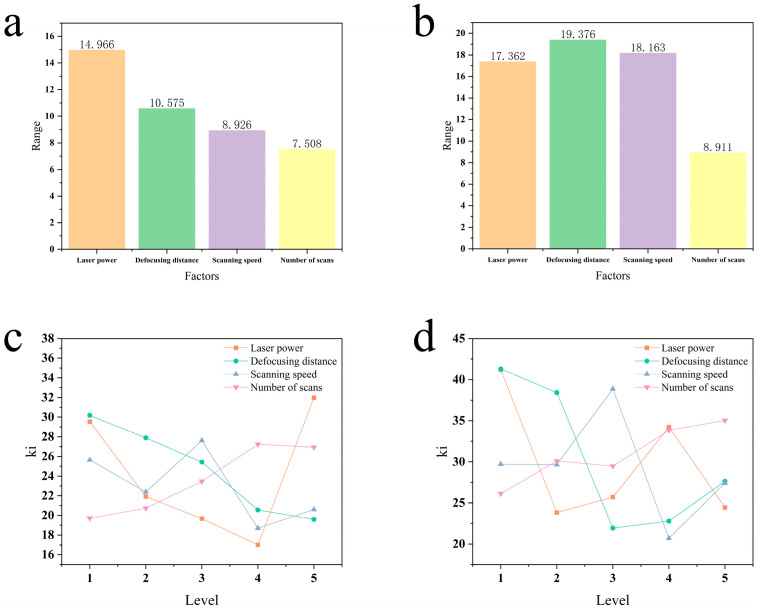
Hole roundness analysis: (**a**) range of entrance hole roundness; (**b**) range of exit hole roundness; (**c**) effect of different levels on entrance hole roundness; (**d**) effect of different levels on exit hole roundness.

Figure 7a presents a bar chart of the maximum deviation values for entrance roundness under each influencing factor. Laser power exerts the most significant effect on roundness deviation. The correlation strength ranking between the four factors and entrance roundness is as follows: A (laser power) > B (defocusing distance) > C (scanning speed) > D (number of scans).

Figure 7c illustrates the effects of different factor levels on the entrance roundness. As shown, laser power is the dominant factor, as it directly determines the stability of the ablation process. When the power is too low, the material removal efficiency becomes unstable, readily causing molten spatter and irregular ablation edges, which results in poor roundness. At a moderate power level where the material is instantaneously vaporized, the heat-affected zone and melting effects are minimized, thus yielding the best roundness. Conversely, excessively high power induces a plasma shielding effect, leading to non-uniform energy distribution along the processing path and a highly irregular entrance shape. Regarding the defocusing distance, roundness improves as the defocus increases; this is because a larger defocus enlarges the spot diameter on the material surface, creating a more gentle and uniform energy distribution. This facilitates more consistent material removal along the entire trepanning path, thereby achieving better roundness. The influence of scanning speed shows a fluctuating trend, with better roundness observed at a moderate speed. This is likely because an excessively high speed provides an insufficient laser-material interaction time, leading to non-uniform melting. In contrast, a speed that is too low can cause the uneven diffusion of molten material, affecting the edge geometry. Roundness deteriorates with an increasing number of scans because as the process goes deeper, laser reflections from the hole wall and the expulsion of plasma and debris become more difficult and erratic. These factors progressively erode the hole entrance edge with each subsequent scan, degrading its roundness. A comprehensive analysis of all factors leads to the optimal combination of A_4_B_5_C_4_D_1_.

Figure 7b presents the range analysis results for exit roundness during the picosecond laser ring cutting and drilling process. The primary factors, in descending order of influence, are B (defocusing distance) > C (scanning speed) > A (laser power) > D (number of scans). Notably, the defocusing distance and scanning speed exert particularly significant effects on exit roundness.

As depicted in Figure 7d, the trends in exit roundness exhibit significant variations at different factor levels. The defocusing distance emerges as the most critical factor. A defocus of 0 mm ensures that the beam, after penetrating the complete material thickness, maintains a relatively well-defined and energy-concentrated spot profile at the exit, which facilitates uniform cutting and yields the optimal roundness. Conversely, an excessively small or large defocus can cause the beam to become severely diverged or result in improper energy deposition by the time it reaches the exit, leading to irregular ablation. The influences of laser power and scanning speed are also pronounced, as they jointly determine the effective energy density delivered to the exit. Given the substantial energy loss during transmission through the hole, insufficient power leads to inadequate energy for achieving stable and continuous material removal at the exit. In contrast, excessive power can excite an overly intense plasma deep within the channel, which, in turn, obstructs energy from reaching the exit. Both scenarios compromise the regularity of the exit hole. In comparison, the number of scans shows minimal overall variation and has a relatively weak influence on exit roundness, primarily serving an auxiliary or fine-tuning role. A comprehensive analysis of all factors identifies the optimal combination as A_2_B_3_C_4_D_1_.

### 3.4. Process Optimization

For the final determination, the comprehensive balancing method was employed. First, each response metric was analyzed independently to rank the correlation strength between influencing factors and metrics and to identify optimal parameter levels; these individual results were then integrated using systematic criteria to derive an overall optimized parameter configuration. As noted above, range analysis and direct comparisons established the correlation strengths and corresponding optimal parameter settings for each factor–metric pair, as shown in Table 8.

To achieve micro-holes with optimal overall performance, it is necessary to balance multiple, often conflicting, quality metrics. The single-objective optimal combinations in Table 8 indicate that the optimal parameter levels vary significantly for different evaluation criteria. For instance, achieving the minimum hole taper requires the A_3_B_5_ combination, while the best hole exit roundness is obtained with A_2_B_3_. Consequently, a simple superposition of these individual optimal levels is not feasible. This study employed the comprehensive balance method to determine the definitive optimal process parameter combination. In precision machining applications, such as film cooling holes in high-temperature alloys, hole taper and roundness are critical geometric indicators that dictate the fluid dynamic characteristics and service performance. Therefore, these metrics were given the highest priority during the optimization process.

The comprehensive balance analysis was conducted as follows. First, for factor D (number of scans), the optimal level was consistently D_1_ across all three key metrics—hole taper, entrance roundness, and exit roundness—demonstrating high consistency. Thus, D_1_ (15 *n*) was unequivocally selected. Second, factor B (defocusing distance) exhibited the most significant influence on hole taper (as evidenced by the most considerable range value), and level B_5_ was optimal for both minimum taper and best entrance roundness. It was, therefore, preferentially selected as B_5_ (+0.2 mm). For factor C (scanning speed), level C_4_ simultaneously optimized both entrance and exit roundness. Although slightly less effective than C_5_ for minimizing taper, C_4_ represents an ideal trade-off for its comprehensive improvement of the hole’s cross-sectional geometry, leading to the selection of C_4_ (27 mm/s). Finally, the optimal levels for factor A (laser power) conflicted. Considering that A_3_ is the optimal level for achieving minimum taper and its value (85%) is positioned between the optimal levels for exit roundness (A_2_, 80%) and entrance roundness (A_4_, 90%), it represents the best compromise. Thus, A3 was determined to be the optimal level.

**Figure 8:**
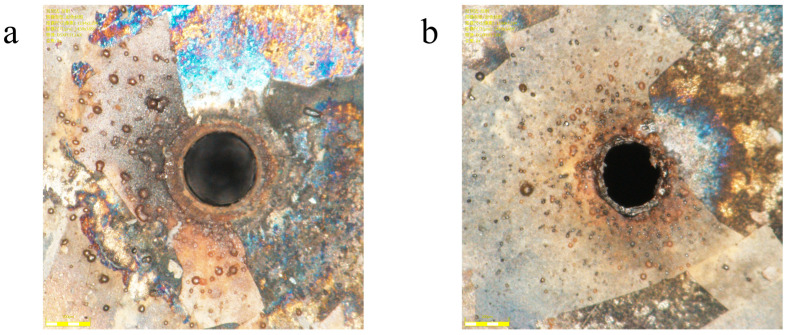
Small holes processed with optimal parameters: (**a**) entrance; (**b**) exit.

In summary, based on the principle of comprehensive balancing, the optimal parameter combination (A_3_B_5_C_4_D_1_) was determined, corresponding to a laser power of 85% (total power: 30 W), defocusing distance of −0.2 mm, scanning speed of 27 mm/s, and number of scans of 15. This combination prioritizes taper optimization while controlling inlet/outlet aperture dimensions and enhances roundness of both apertures, thereby improving overall micro-hole processing quality. The holes produced are shown in Figure 8. The entrance diameter is 325.838 µm, the exit diameter is 289.773 µm, the taper is 2.06° (0.036 rad), the entrance roundness is 6.196 µm, and the exit roundness is 22.057 µm, and there is no accumulation of molten material on the hole surface. This result, free from significant melt-related defects, demonstrates the advantage of the picosecond laser regime, which facilitates a more deterministic ablation process with minimal thermal side effects compared to longer pulse durations [22].

## 4. Discussion

### 4.1. Comprehensive Optimization Strategy

A key contribution of this study is the application of the “Comprehensive Balancing Method” for process optimization. As demonstrated in Table 8, the optimal parameter levels for different quality metrics often conflict. For instance, the parameter combination that yields the optimal exit diameter (A_1_B_1_) directly conflicts with the combination required for the minimum taper (A_3_B_5_). Consequently, simply aggregating the optimal levels for individual metrics is impractical for real-world applications.

Our optimization strategy, therefore, prioritized taper and roundness. This decision was directly driven by the functional requirements of critical applications, such as film cooling holes in aero-engine turbine blades. In such applications, the hole geometry directly governs the fluid dynamic characteristics and the service life of the component, making taper and roundness paramount indicators. By balancing secondary metrics to maximize performance in these key areas, we ultimately identified A_3_B_5_C_4_D_1_ as the globally optimal combination.

### 4.2. Comparative Analysis with Existing Research

To evaluate the novelty and advantages of our findings, the optimized results from this study were benchmarked against the existing literature in the field of laser drilling, as shown in Table 9. It is essential to note that a direct quantitative comparison is challenging due to variations in experimental conditions across studies, such as material thickness and the type of laser system used. Nevertheless, comparing key quality metrics demonstrates the advantages of the optimized process developed in this work.

Several important conclusions can be drawn from this comparison:

1. Advantages over Millisecond Lasers: Compared to conventional millisecond laser drilling [23], the picosecond process used in this study achieved a significantly smaller taper and superior surface quality, as evidenced by the clean exit hole shown in Figure 8. This is a direct result of the “cold ablation” mechanism characteristic of ultrashort pulses, where material removal is completed before significant thermal diffusion can occur. This fundamentally suppresses the melt pool effects and thermal damage inherent to long-pulse laser processing.
2. Competitive Performance against Femtosecond Lasers: Although femtosecond lasers [16] can achieve nearly perfect “athermal” ablation, our picosecond laser process presents a highly competitive alternative. We achieved excellent taper control with minimal thermal damage. Critically, picosecond laser systems typically offer higher average power and greater cost-effectiveness, enabling significantly higher processing throughput than their femtosecond counterparts.

## 5. Conclusions

Based on the results of the experiment, the following conclusions can be drawn:

1. Scanning speed and laser power predominantly affect the entrance and exit diameters of micro-holes, whereas other parameters exhibit lesser influence. The defocusing distance directly correlates with micro-hole taper. The laser power demonstrates the strongest correlation with entrance roundness, while the defocusing distance, number of scans, and laser power collectively influence exit roundness. The number of scans exhibits negligible correlation with the entrance/exit diameters, taper, or roundness characteristics of produced micro-holes.
2. For entrance diameter optimization, the optimal parameter combination is A_4_B_3_C_4_D_5_, with a laser power of 90% (30 W), defocusing distance of 0 mm, scanning speed of 27 mm/s, and number of scans of 55. Regarding the exit diameter, the combination A_1_B_1_C_4_D_3_ delivers optimal results: a laser power of 75% (30 W), defocusing distance of −0.2 mm, scanning speed of 27 mm/s, and number of scans of 35. Taper optimization is achieved with combination A_3_B_5_C_5_D_1_: a laser power of 85% (30 W), defocusing distance of 0.2 mm, scanning speed of 30 mm/s, and number of scans of 15. For entrance roundness, the combination A_4_B_5_C_4_D_1_ is optimal: a laser power of 90% (30 W), defocusing distance of 0.2 mm, scanning speed of 27 mm/s, and number of scans of 15. Finally, exit roundness optimization requires the combination A_2_B_3_C_4_D_1_: a laser power of 80% (30 W), defocusing distance of 0 mm, scanning speed of 27 mm/s, and number of scans of 15.
3. The optimal parameter levels vary significantly depending on the specific quality metric being evaluated. In applications involving film cooling holes in superalloys, hole taper and roundness are critical geometric indicators that determine the fluid dynamic characteristics and in-service performance; therefore, they should be given the highest priority during the optimization process. By employing range analysis and a comprehensive balancing method to evaluate the multiple hole quality metrics, a final, globally optimal parameter combination was identified as A_3_B_5_C_4_D_1_, corresponding to a laser power of 90%, a defocusing distance of +0.2 mm, a scanning speed of 27 mm/s, and 15 scans.

## Figures and Tables

**Table 1:** The chemical composition of the titanium alloy TC4.

Composition	Ti	Al	V	Fe
Mass fraction/%	Allowance	6.5~6.8	4.2~4.5	0.3
Composition	C	N	H	O
Mass fraction/%	0.1	0.05	0.015	0.2

**Table 2:** Orthogonal factor level.

Level	Factor			
	A Laser Power (%)	B Defocusing Distance (mm)	C Scanning Speed (mm/s)	D Number of Scans (*n*)
1	75	−0.2	18	15
2	80	−0.1	21	25
3	85	0	24	35
4	90	0.1	27	45
5	95	0.2	30	55

**Table 3:** Average response and range of entrance diameter.

Level	A Laser Power (%)	B Defocusing Distance (mm)	C Scanning Speed (mm/s)	D Number of Scans (*n*)
k1	327.7965	332.9658	331.7113	332.3041
k2	331.668	335.7154	303.6404	317.6234
k3	339.2347	311.4376	329.0273	319.8893
k4	314.6515	335.664	253.2778	320.3007
k5	323.1115	318.8621	264.0036	316.2983
R	24.58315	24.27783	78.43353	16.00585

**Table 4:** Average response and range of exit diameter.

Level	A Laser Power (%)	B Defocusing Distance (mm)	C Scanning Speed (mm/s)	D Number of Scans (*n*)
k1	272.9397	273.5901	290.1488	288.5664
k2	284.9136	278.2018	292.3072	288.6472
k3	306.1691	289.9006	284.1174	286.4271
k4	294.2274	300.674	232.7996	291.3311
k5	288.4133	302.811	237.5614	287.3594
R	33.2294	29.2209	59.50765	4.904

**Table 5:** Average response and range of taper.

Level	A Laser Power (%)	B Defocusing Distance (mm)	C Scanning Speed (mm/s)	D Number of Scans (*n*)
k1	6.274851	6.79256	4.75646	5.00718
k2	5.351858	6.579758	4.764565	5.037405
k3	3.786666	4.213454	5.138486	5.550425
k4	4.05852	4.006434	4.091225	5.036334
k5	5.717751	3.559572	3.027081	5.032606
R	2.488186	3.232988	2.111404	0.543245

**Table 6:** Average response and range of entrance hole roundness.

Level	A Laser Power (%)	B Defocusing Distance (mm)	C Scanning Speed (mm/s)	D Number of Scans (*n*)
k1	29.5468	30.1784	25.6492	19.7216
k2	21.9214	27.891	22.38	20.7368
k3	19.6728	25.4246	27.6158	23.4764
k4	17.0136	20.5604	18.69	27.2294
k5	31.9798	19.6034	20.602	26.9362
R	14.9662	10.575	8.9258	7.5078

**Table 7:** Average response and range of exit hole roundness.

Level	A Laser Power (%)	B Defocusing Distance (mm)	C Scanning Speed (mm/s)	D Number of Scans (*n*)
k1	41.1982	41.3	29.6976	26.1204
k2	23.8358	38.42	29.6526	30.1074
k3	25.6982	21.924	38.8708	29.4642
k4	34.23	22.78	20.708	33.8316
k5	24.4476	27.6326	27.4046	35.031
R	17.3624	19.376	18.1628	8.9106

**Table 8:** Optimal parameter combinations for each factor.

Hole entrance diameter	Range	24.58315	24.27783	78.43353	16.00585
	Optimal factor	A_4_	B_3_	C_4_	D_5_
Hole exit diameter	Range	33.2294	29.2209	59.50765	4.904
	Optimal factor	A_1_	B_1_	C_4_	D_3_
Hole taper	Range	2.488186	3.232988	2.111404	0.543245
	Optimal factor	A_3_	B_5_	C_5_	D_1_
Hole entrance roundness	Range	14.9662	10.575	8.9258	7.5078
	Optimal factor	A_4_	B_5_	C_4_	D_1_
Hole exit roundness	Range	17.3624	19.376	18.1628	8.9106
	Optimal factor	A_2_	B_3_	C_4_	D_1_

**Table 9:** Comparison of laser drilling quality.

Source	Laser Type	Material and Thickness	Taper
N D Bahar et al. [23]	Millisecond laser	TC4, 1.0 mm	1.8°
P. Deepu et al. [16]	Femtosecond laser	TC4, 0.5 mm	0.032 rad
This study	Picosecond laser	TC4, 0.5 mm	2.06° (0.036 rad)

## Data Availability

The original contributions presented in this study are included in the article. Further inquiries can be directed to the corresponding authors.

## References

[1] Yuan C.G., Pramanik A., Basak A.K., Prakash C., Shankar S. (2021). Drilling of titanium alloy (Ti6Al4V)—A review. Mach. Sci. Technol..

[2] Li Z., Wei X., Guo Y., Sealy M.P. (2015). State-of-art, challenges, and outlook on manufacturing of cooling holes for turbine blades. Mach. Sci. Technol..

[3] Liu W., Liu S., Wang L. (2019). Surface modification of biomedical titanium alloy: Micromorphology, microstructure evolution and biomedical applications. Coatings.

[4] Jia X., Chen Y., Liu L., Wang C., Duan J. (2022). Advances in laser drilling of structural ceramics. Nanomaterials.

[5] Chen H., Dechun G., Tong C., Daoli L., Chen B. (2018). Second-derivative laser-induced fluorescence spectroscopy combined with chemometrics for authentication of the adulteration of camellia oil. CyTA-J. Food.

[6] Feng J., Zhang R., Dabbour M., Mintah B.K., Gao X., He R., Ma H. (2023). Enhancing acid production of Acetobacter pasteurianus by laser and intense pulsed light mutagenesis and its molecular mechanism based on transcriptomic analysis. LWT.

[7] Xia K., Ren N., Lin Q., Yang H. (2023). Femtosecond laser drilling in superalloy with water-based magnetic assistance. Opt. Commun..

[8] Zhang F., Wang J., Wang X., Zhang J., Hayasaki Y., Kim D., Sun S. (2021). Experimental study of nickel-based superalloy IN792 with femtosecond laser drilling method. Opt. Laser Technol..

[9] Lu F., Ruan S., Wang Y., Li Y., Ma F., Ma H. (2024). Unveiling underlying mechanism of combined He–Ne laser and UV mutagenesis in Bacillus subtilis CICC 21927: A transcriptomic analysis. Food Biosci..

[10] Tian X.Y., Aheto J.H., Dai C., Ren Y., Bai J.W. (2021). Monitoring microstructural changes and moisture distribution of dry-cured pork: A combined confocal laser scanning microscopy and hyperspectral imaging study. J. Sci. Food Agric..

[11] Zhu Y.D., Zou X.B., Shi J.Y., Zhao J.W., Huang X.W. (2017). Observation of the Oil Content of Fried Lotus (Nelumbo nucifera Gaertn.) Root Slices by Confocal Laser Scanning Microscopy Based on Three-Dimensional Model. J. Food Process. Preserv..

[12] Sun J., Zhang D., Jing X., Zheng S., Sun H. (2023). Experimental investigation and optimization on trepanning drilling in K24 superalloy by femtosecond laser via orthogonal experiment. Int. J. Adv. Manuf. Technol..

[13] Amiri S., Khajehzadeh M., Razfar M.R. (2020). Magnetic field and ultrasonic aided laser drilling effect on Ti6Al4V microstructural characteristics. Mater. Manuf. Process..

[14] Ku K.A.I.F.B., Halil A.B.M., Ishak M.B., Yusof M.F.B.M., Shah L.H.B.A. (2024). Effect of Laser Process Loops on the Hole Diameter and Hole Formation of Laser Micro Drilling on TC4. J. Phys. Conf. Ser..

[15] Saravanan M., Raja V.B., Palanikumar K., Vaidyaa P., Sundar S., Prakash M.S. (2021). Laser drilling parameter optimization for Ti6Al4v alloy. Mater. Today Proc..

[16] Deepu P., Jagadesh T., Duraiselvam M. (2023). Investigations into morphology and surface integrity of micro-hole during femtosecond laser drilling of titanium alloy. J. Braz. Soc. Mech. Sci. Eng..

[17] Bupesh Raja V., Sonawwanay P.D., Duraivelu K., Alphonse M., Muhammad N., Lazar P. Optimization of Cutting Parameters of Laser Drilling on Ti6Al4V Alloy Using ANN. Proceedings of the International Conference on Advanced Materials Manufacturing and Structures.

[18] Shin J., Mazumder J. (2017). Shallow angle drilling of inconel 718 using a helical laser drilling technique. J. Manuf. Sci. Eng..

[19] Du T., Liang X., Yu Y., Zhou L., Cai Z., Wang L., Jia W., Pan X. (2023). Optimization of femtosecond laser drilling process for DD6 single crystal alloy. Metals.

[20] Schulz W., Eppelt U., Poprawe R. (2013). Review on laser drilling I. Fundamentals, modeling, and simulation. J. Laser Appl..

[21] Holder D., Weber R., Graf T., Onuseit V., Brinkmeier D., Förster D.J., Feuer A. (2021). Analytical model for the depth progress of percussion drilling with ultrashort laser pulses. Appl. Phys. A.

[22] Webster P.J., Yu J.X., Leung B.Y., Anderson M.D., Yang V.X., Fraser J.M. (2010). In situ 24 kHz coherent imaging of morphology change in laser percussion drilling. Opt. Lett..

[23] Bahar N., Marimuthu S., Yahya W. (2016). Pulsed Nd: YAG laser drilling of aerospace materials (Ti-6Al-4V). IOP Conf. Ser. Mater. Sci. Eng..

